# Mechanical nanosurgery approach: assistance to overcome the chemotherapy resistance of glioblastoma

**DOI:** 10.1002/mco2.373

**Published:** 2023-09-17

**Authors:** Qi Chen, Di Wu, Zhong Chen

**Affiliations:** ^1^ Key Laboratory of Neuropharmacology and Translational Medicine of Zhejiang Province School of Pharmaceutical Sciences Zhejiang Chinese Medical University Hangzhou China

1

In a recent publication in Science Advances, Sun et al. reported the utility of magnetic carbon nanotubes (mCNTs) that efficiently be activated by the spatiotemporally controlled rotating magnetic field and then induce glioblastoma (GBM) cell death through the produced mechanical work, representing a promising approach for treating chemoresistant GBM or those cannot be readily removed by conventional surgery.^1^

GBM refers to the most aggressive cancer in the brain. The standard of treatment for GBM is surgery with daily radiation and oral chemotherapy. Temozolomide (TMZ) is a chemo drug that obtained the approval from US Food and Drug Administration (FDA) and is commonly used to treat GBM. Unfortunately, gradually, TMZ lost control of GBM due to the resistance generated. Finally, this phenomenon leads to chemotherapy failure of approximately half of the glioblastoma showing tumor relapse and patient mortality. It is urgent to find an approach aiming at chemoresistant GBM. Some studies focus on selected proteins or biochemical pathways to modulate the microenvironment for improving the chemo‐sensitivity of GBM, however, GBM is heterogeneous in both biochemical signaling and cellular composition levels, which means this method type lacks universality.^2^

Therefore, Sun's group looked for other ways and found that GBM cells are mechanosensitive.^3^ They first hypothesized magnetic nanomaterial‐based mechanical treatment could target and break the integrity of GBM cells through mechanical perturbation. They developed biocompatible mCNTs which not only have carbon surface but also have iron particles filled in the cavity. Further, the authors functionalized the carbon surface of mCNTs with anti‐CD44 antibodies to enhance chemotherapy resistance GBM enrichment and retention. mCNT and mCNT^CD44^ were designed as a result (Figure 1A). Next, a custom‐built magnetic field generation system was used to place a petri dish containing the co‐cultured GBM stem cells or patient‐derived GBM cell lines with mCNTs or mCNT^CD44^ (Figure 1B). The results proved in vitro that magnetic field treatment caused an obvious cell death rate. GBM‐bearing mice were used to study magnetic field treatment in vivo, including the distribution of mCNT, magnetic field control, and the impact of the treatment on tumor growth and survival, via a magnetic field generation system, which includes an editable function generator, a custom‐designed electric current amplifier, four magnetic conductors with magnetic cores, and an oscilloscope (Figure 1C). In this section of exploration, they added primary TMZ resistance models using CRISPR‐induced knockout of MSH6 of GBM cells and treatment‐induced TMZ resistance models using increased dosages of TMZ to treat and select the live GBM cells. mCNT^CD44^ performed well in all aspects of the involved studies.

**FIGURE 1:**
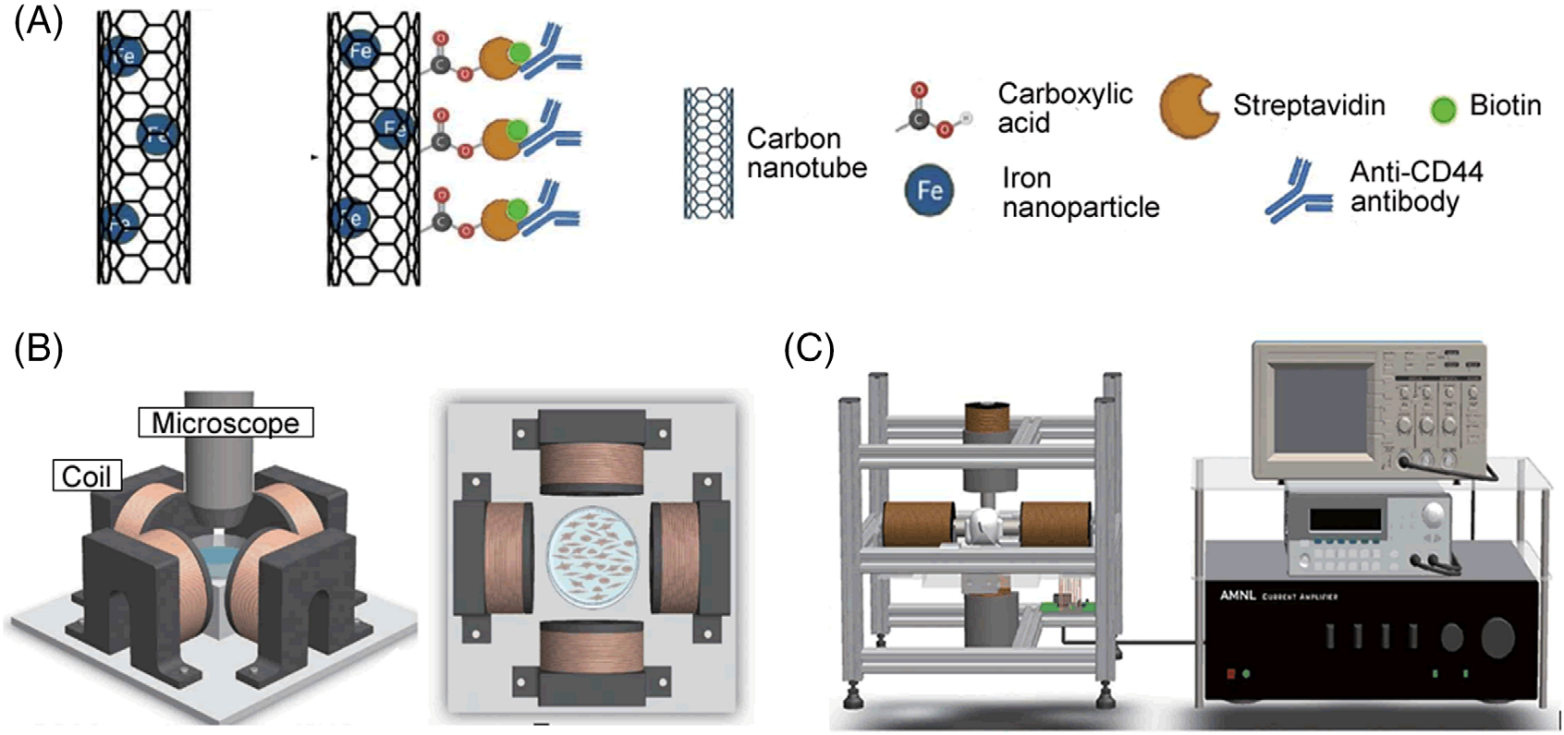
Illustration of magnetic nanomaterials and the magnetic field generation system used in mechanical nanosurgery. (A) The structure of magnetic carbon nanotubes (mCNTs) and anti‐CD44 functionalized mCNTs (mCNT^CD44^). (B) Magnetic coil system for generating rotating magnetic fields. (C) Magnetic field generation system for treating glioblastoma (GBM)‐bearing mice. Copyright Permission.^1^

One particularly important advance made by Sun et al. is that they examined the Cancer Genome Atlas low‐grade glioma‐GBM and noticed CD44 ^−^positive glioma cells confer TMZ resistance. Hence, in their strategy, CD44 was chosen for mCNTs functionalization to increase the recognition of chemoresistant GBM cells. Impressively, they have paid attention to the toxicity of mCNTs. It was reported that the toxicity of these inorganic carriers mainly comes from the difficulty in degradation, inducing oxidative stress, generating reactive oxygen species, leading to inflammation, and so on.^4^ Similarly, the interaction of brain cells and CNTs could induce microglia and astrocytes to release various mediators/chemicals which may result in neurotoxicity in the brain.^5^ To maximize the biocompatibility of their mCNTs, they used citric acid instead of a strong acid to yield openings in bonds and to functionalize the exterior of mCNT with a carboxyl group, followed by the connection of streptavidin‐amine, biotin, and anti‐CD44 antibody step by step, which was gentler and safer. Besides, they compared the decorative site of iron particles either inside the nanotubes or on the surface and chose the inside manner because of the lower cytotoxicity. The carbon surface could serve as a barrier preventing the contact of iron particles and cells while the iron particle could serve as the subject commanded by a magnetic field. Overall, applying mCNT^CD44^ to cancer cells more accurately through a magnetic field can reduce contact with normal cells and then reduce potential toxicity.

Of note, the principle was to transfer rotational energy from mobilizing mCNTs under the magnetic field. They used the uniform magnetic field and 2D cell culture model for the cell study, which provided limited information and was unable to correspond with the subsequent animal experiments involving an alternating magnetic field. Therefore, we think 3D tumor spheroids models are highly recommended for improving the manuscript. Principally, when compared to other non‐invasive treatments, such as tumor‐treating fields, focused ultrasound, and so on, their mechanical nanosurgery approach is not associated with a specific biochemical process. By utilizing both the top‐down and side views in bioluminescence images, the GBM region was fixed through coordinate transformation. In this way, mCNT could be placed in the tumor precisely and the side effects on nontumoral brain tissues could be reduced by relying on a tumor region‐focused magnetic field. Hence, the mechanical nanosurgery approach exhibits great advantages in studying drug‐resistant tumors, especially the TMZ, inducing DNA damage. However, for those dispersed or multiple metastatic tumors, tumor region‐focused magnetic fields are hard to build, only uniform rotating magnetic fields can be used. How to optimize the mCNT platform becomes a main thinking direction. Researchers are supposed to search for the characteristics of those dispersed or multiple metastatic tumors to make the platform reach the target area relying on other means instead of magnetic control. After reaching the target area, the mechanical nanosurgery approach based on uniform rotating magnetic fields could help.

In summary, Sun and colleagues advance the magnetic field–actuated nanomaterials by constructing mCNT and mCNT^CD44^, and the systematic strategies to concentrate the energy of the magnetic field to avoid damage to normal brain tissue. The strategy is relatively facile, allowing fast and wide application not only in the chemotherapy resistance of glioblastoma but also in debulking multifocal tumors that cannot be readily removed by conventional surgery in the central nervous system. Their study boosts future research on the utility of mechanical nanosurgery. The mechanical nanosurgery approach can be further optimized according to actual needs. Their well‐designed mCNT platform is expandable and has a large surface area that can be functionalized with various functional groups, which is not limited to improving the tumor‐homing ability but also could be conjugated with photothermal therapy, immunotherapy, and so on, representing promising avenues. Other magnetic particles are also worth exploring in the field of mechanical nanosurgery, such as superparamagnetic iron oxide, nickel, and cobalt. What's more, with future developments of hybrid technologies, the mechanical nanosurgery approach can combine with other systems, not only the referred fluorescence imaging systems in the original research article, for example, surface‐enhanced Raman spectroscopy, magnetic particle imaging systems, or magnetic resonance imaging systems, developing more safe and reliable operation for use. We believe the applications can gradually expand to other intractable diseases.

## AUTHOR CONTRIBUTIONS

Qi Chen wrote the manuscript and prepared the figure. Di Wu and Zhong Chen have read and discussed this manuscript. All authors have read and approved the final manuscript.

## CONFLICT OF INTEREST STATEMENT

The authors declare no conflict of interest.

## ETHICS STATEMENT

Not applicable.

## Data Availability

Not applicable.
